# Management of hypopituitarism: a perspective from the Brazilian Society of Endocrinology and Metabolism

**DOI:** 10.20945/2359-3997000000335

**Published:** 2021-02-25

**Authors:** Heraldo Mendes Garmes, César Luiz Boguszewski, Paulo Augusto Carvalho Miranda, Manoel Ricardo Alves Martins, Silvia Regina Correa da Silva, Julio Zaki Abucham, Nina Rosa de Castro Musolino, Lucio Vilar, Luiz Henrique Corrêa Portari, Mônica Roberto Gadelha, Leandro Kasuki, Luciana Ansaneli Naves, Mauro Antônio Czepielewski, Tobias Skrebsky de Almeida, Felipe Henning Gaia Duarte, Andrea Glezer, Marcello Delano Bronstein

**Affiliations:** 1 Universidade Estadual de Campinas Faculdade de Ciências Médicas Departamento de Clínica Médica Campinas SP Brasil Unidade de Neuroendocrinologia, Divisão de Endocrinologia e Metabologia, Departamento de Clínica Médica, Faculdade de Ciências Médicas, Universidade Estadual de Campinas (Unicamp), Campinas, SP, Brasil; 2 Universidade Federal do Paraná Departamento de Clínica Médica Serviço de Endocrinologia e Metabologia Curitiba PR Brasil Serviço de Endocrinologia e Metabologia, Departamento de Clínica Médica, Universidade Federal do Paraná (SEMPR), Curitiba, PR, Brasil; 3 Santa Casa de Belo Horizonte Serviço de Endocrinologia e Metabologia Belo Horizonte MG Brasil Serviço de Endocrinologia e Metabologia, Santa Casa de Belo Horizonte, Belo Horizonte, MG, Brasil; 4 Universidade Federal do Ceará Faculdade de Medicina Departamento de Medicina Clínica Fortaleza CE Brasil Departamento de Medicina Clínica, Faculdade de Medicina da Universidade Federal do Ceará, Fortaleza, CE, Brasil; 5 Universidade Federal de São Paulo Escola Paulista de Medicina Divisão de Endocrinologia e Metabolismo São Paulo SP Brasil Unidade de Neuroendocrinologia, Divisão de Endocrinologia e Metabolismo, Escola Paulista de Medicina, Universidade Federal de São Paulo (EPM-Unifesp), São Paulo, SP, Brasil; 6 Universidade de São Paulo Faculdade de Medicina Hospital das Clínicas São Paulo DP Brasil Unidade de Neuroendocrinologia, Divisão de Neurocirurgia Funcional, Hospital das Clínicas, Faculdade de Medicina da Universidade de São Paulo, São Paulo, DP, Brasil; 7 Universidade Federal de Pernambuco Hospital das Clínicas Serviço de Endocrinologia Recife PE Brasil Serviço de Endocrinologia, Hospital das Clínicas da Universidade Federal de Pernambuco, Recife, PE, Brasil; 8 Universidade Federal do Rio de Janeiro Hospital Universitário Clementino Fraga Filho Centro de Pesquisa de Neuroendocrinologia Rio de Janeiro RJ Brasil Unidade de Neuroendocrinologia, Instituto Estadual do Cérebro Paulo Niemeyer, Centro de Pesquisa de Neuroendocrinologia, Hospital Universitário Clementino Fraga Filho, Universidade Federal do Rio de Janeiro, Rio de Janeiro, RJ, Brasil; 9 Universidade de Brasília Faculdade de Medicina Serviço de Endocrinologia Brasília DF Brasil Serviço de Endocrinologia, Faculdade de Medicina da Universidade de Brasília, Brasília, DF, Brasil; 10 Universidade Federal do Rio Grande do Sul Faculdade de Medicina Hospital de Clínicas de Porto Alegre Porto Alegre RS Brasil Serviço de Endocrinologia, Hospital de Clínicas de Porto Alegre; Faculdade de Medicina, Universidade Federal do Rio Grande do Sul, Porto Alegre, RS, Brasil; 11 A.C. Camargo Cancer Center Serviço de Endocrinologia Oncológica São Paulo SP Brasil Serviço de Endocrinologia Oncológica, A.C. Camargo Cancer Center, São Paulo, SP, Brasil; 12 Universidade de São Paulo Faculdade de Medicina Hospital das Clínicas São Paulo SP Brasil Unidade de Neuroendocrinologia, Laboratório de Endocrinologia Celular e Molecular LIM-25, Divisão de Endocrinologia e Metabolismo, Hospital das Clínicas, Faculdade de Medicina da Universidade de São Paulo, São Paulo, SP, Brasil

**Keywords:** Hypopituitarism, central hypothyroidism, secondary adrenal insufficiency, central hypogonadism and GH deficiency

## Abstract

Hypopituitarism is a disorder characterized by insufficient secretion of one or more pituitary hormones. New etiologies of hypopituitarism have been recently described, including head trauma, cerebral hemorrhage, and drug-induced hypophysitis. The investigation of patients with these new disorders, in addition to advances in diagnosis and treatment of hypopituitarism, has increased the prevalence of this condition. Pituitary hormone deficiencies can induce significant clinical changes with consequent increased morbidity and mortality rates, while hormone replacement based on current guidelines protects these patients. In this review, we will first discuss the different etiologies of hypopituitarism and then address one by one the clinical aspects, diagnostic evaluation, and therapeutic options for deficiencies of TSH, ACTH, gonadotropin, and GH. Finally, we will detail the hormonal interactions that occur during replacement of pituitary hormones.

## INTRODUCTION

Hypopituitarism is a heterogeneous disease characterized by insufficient secretion of one or more pituitary hormones due to genetic or acquired causes (1). The only available epidemiological data estimating the frequency of hypopituitarism in the adult population derived from a Spanish study published in 2001 showing a prevalence of 455 cases per million inhabitants and an incidence of 42.1 cases per million inhabitants per year (2,3). If we consider these numbers to be true for our population, Brazil has approximately 100,000 patients with hypopituitarism and roughly 8,500 new cases per year. However, these numbers certainly underestimate the frequency of hypopituitarism, which has increased over the last years due to recognition of new etiologies, such as cerebral hemorrhage and head trauma, emergence of new etiologies, such as drug-induced hypophysitis, and improvements in diagnostic tools (1). Hypopituitarism has been associated with increased mortality, particularly due to cardiovascular and cerebrovascular diseases (4). Two recent meta-analyses involving observational studies have confirmed such increased mortality, with higher rates observed especially in women and in patients of younger age at diagnosis (5,6). Nevertheless, new concepts in the pathophysiology of hormonal deficiencies, recent advances in diagnostic tools, and the emergence of new formulations for hormone replacement have significantly contributed to a reduction in morbidity and mortality rates in these patients (7-10). This narrative review provides a guide for the management of patients with hypopituitarism in Brazil, considering as much as possible regional differences and health care disparities.

## ETIOLOGY

Hypopituitarism is a consequence of disorders that compromise the secretory function of the anterior pituitary or interfere with the hypothalamic secretion of anterior pituitary-releasing hormones. Hypopituitarism may be secondary to genetic defects, congenital abnormalities, or acquired lesions, such as tumors, vascular abnormalities, trauma, or inflammatory, infiltrative, and infectious diseases. In the pediatric population, the most frequent causes of hypopituitarism are genetic or congenital disorders affecting the hypothalamic-pituitary region, usually associated with mutations or low expression of transcription factors responsible for pituitary development, alteration in hypothalamic hormone receptors, structural defects, or mutations in pituitary hormones or their subunits (11). On the other hand, acquired causes are more prevalent in adulthood. Despite that, pituitary tumors and their treatment with surgery and/or radiotherapy account for up to two-thirds of the cases in most literature series. Indeed, in a recent single-center Brazilian study involving 99 adult patients with hypopituitarism, around half of the patients had nontumoral causes of hypopituitarism, while FSH/LH, GH, TSH, and ACTH deficiencies were present in 99%, 98.6%, 96%, and 81.8% of them, respectively (12). Of note, Sheehan’s syndrome – necrosis of the pituitary gland occurring during or soon after labor – continues to be a common cause of hypopituitarism in women in developing regions of the world (13). Aneurysmal subarachnoid hemorrhage is another nontumoral etiology associated with pituitary deficiency (14). A study carried out in Belo Horizonte, Brazil, observed some degree of pituitary dysfunction in 59% of 66 consecutive patients evaluated in the first 15 days after aneurysmal subarachnoid hemorrhage (15). However, the prevalence of hypopituitarism in this group of patients decreases in the long term, suggesting that some deficiencies are temporary and may improve over time (16).

The prevalence of hypopituitarism following traumatic brain injury (TBI) is extremely variable across studies, with percentages ranging from 16% to 69% (owing mainly to differences in study populations, severity of trauma, time of evaluation, and laboratory criteria used to define pituitary deficiency), while some authors consider these rates to be overestimated (14,17,18). Brazilian investigators have studied the association between low LH and testosterone levels with morbidity and mortality during the acute phase of severe TBI, but the role of these hormones as prognostic factors is still uncertain (19). Similarly, sports-related TBI, comprising continuous or acute trauma in professional athletes, amateur sporting, or even during recreational activities, may also result in pituitary dysfunction that is commonly neglected and undiagnosed (20).

Hypophysitis, another frequently underdiagnosed cause of hypopituitarism, is related to an inflammatory process of the pituitary and may be classified as primary or secondary, depending on its etiology. Primary hypophysitis has a prevalence in the population of approximately 0.2–0.88% and an annual incidence of 1/9,000,000 (21). The most common form of hypophysitis is lymphocytic or autoimmune, corresponding to approximately 72% of the cases (22,23). Secondary hypophysitis is related to inflammatory (sarcoidosis, granulomatosis with polyangiitis), infectious (tuberculosis, syphilis, fungal infections), or infiltrative (hemochromatosis, amyloidosis, Langerhans-cell histiocytosis) diseases, or may be drug-related, as observed with two classes of immune checkpoint inhibitors, namely, cytotoxic T lymphocyte associated antigen (CTLA-4) and programmed cell death protein 1 receptor (PD-1) inhibitors (21,24,25). Hypophysitis affects 11–12% of the patients treated with CTLA-4 inhibitors (ipilimumab and tremelimumab) and is more prevalent in men. In contrast, hypophysitis is less common during therapy with PD-1 inhibitors (nivolumab and pembrolizumab) and is rarely observed with PD-L1-targeted drugs (atezolizumab, avelumab) (26). PD-1/PD-L1 interaction may regulate apoptosis, while PD-1/PD-L1 blockade may result in positive activation of the immune system with consequent inhibition of tumor growth (21,27). Early recognition and appropriate management of immune-mediated hypophysitis are important to initiate pituitary hormone replacement without interrupting cancer treatment (Tables 1 and 2) (28).

**Table 1:** Main etiologies of congenital hypopituitarism (adapted from Reference^28^)

Etiologies of Congenital Hypopituitarism	
Mutation	Hormonal deficiency
***Associated with syndromes***	
Kallmann Syndrome	FSH, LH
Prader-Willi Syndrome	FSH, LH
Laurence-Moon-Biedl Syndrome	FSH, LH
***Receptor***	
GHRH Receptor	GH
CRH Receptor	ACTH
GnRH Receptor	FSH, LH
GPR54	FSH, LH
TRH Receptor	TSH
Leptin Receptor	FSH, LH
***Structural***	
Pituitary Aplasia	Any hormonal deficiency
Pituitary Hypoplasia	Any hormonal deficiency
CNS Tumor, Encephalocele	Any hormonal deficiency
***Transcription factor defects***	
HESX1	GH, PRL, TSH, LH, FSH, ACTH
SOX2/3	GH, PRL, TSH, LH, FSH, ACTH
LHX3/4	GH, PRL, TSH, LH, FSH
PITX2	GH
PROP1	GH, PRL, TSH, LH, FSH, ACTH
POU1F1	PRL, GH, TSH
IGSF1	PRL, GH, TSH
TBX19	ACTH
NR5A1	LH, FSH
NR0B1	LH, FSH
***Hormone mutation***	
GH	GH
GH biologically inactive	GH
FSHβ	FSH
LHβ	LH
POMC	ACTH
TSHβ	TSH
Leptin	FSH, LH
PCI	ACTH,FSH,LH
Kisspeptin	FSH,LH

**Table 2:** Main etiologies of acquired hypopituitarism (adapted from Reference 28)

Etiologies of Acquired Hypopituitarism			
***Traumatic***		***Tumors***	
Prior surgery		Pituitary adenoma	
Radiotherapy		Empty sella	
Traumatic brain injury		Parasellar tumors or cysts	
***Infiltrating/Inflammatory***			Rathke’s cyst
Primary hypophysitis			Dermoid cyst
	Lymphocytic		Meningioma
	Granulomatous		Germinoma
	Xanthomatous		Chordoma
Secondary hypophysitis			Ependymoma
	Sarcoidosis		Glioma
	Granulomatosis with polyangiitis		Pinealoma
	Langerhans cell histiocytosis	Craniopharyngioma	
	Infection	Hypothalamic hamartomas	
Hemochromatosis		Gangliocytoma	
Amyloidosis		Metastasis	
Pituitary abscess		Hematological malignancies	
PIT-1 antibody			Leukemias
			Lymphomas
***Associated with infections***		***Functional***	
Tuberculosis		Nutritional	
Syphilis		Excessive physical activity	
*Pneumocystis jirovecii*		Serious diseases	
Fungi (histoplasmosis, aspergillosis)			Acute diseases
Parasites (toxoplasmosis)			Chronic renal failure
Viruses (cytomegalovirus)			Chronic hepatic failure
		Hormonal	
			Hyperprolactinemia
			Primary hypothyroidism
***Vascular***		***Drug-induced dysfunctions***	
Related to pregnancy (Sheehan’s syndrome)		Glucocorticoids	
		Dopamine agonists	
Aneurismal subarachnoid hemorrhage		Somatostatin analogs	
Pituitary apoplexy		Retinoids	
Diabetes		Anti-PD-1 monoclonal antibodies	
Hypotension		Anti-CTLA-4 monoclonal antibodies	
Arteritis		Opioids	
		Anticonvulsants	

## CLINICAL MANIFESTATIONS OF HYPOPITUITARISM

Clinical symptoms of hypopituitarism vary greatly depending on the cause, the age of the patient and speed of onset, affected pituitary hormones, and magnitude of hormone deficiency. The symptoms usually develop insidiously in adults, begin up to several years before diagnosis, and are generally nonspecific, including weakness, tiredness, lethargy, increased sensitivity to cold, discomfort, appetite loss, and weight loss or gain. Most patients with hypopituitarism have multiple pituitary hormone deficiencies, and it is challenging to assign specific signs and symptoms to a single hormone deficiency (1,11,29,30). Hypopituitarism has a variable dynamic throughout its development and follow-up, characterized by a complete or sequential loss of pituitary function, which is usually permanent, although transient deficiencies with recovery years after the initial event may occur (1,14,17,30). The sensitivity of the different pituitary hormones to pathological damage is variable. The usual sequential pattern of hormonal failure is loss of secretion of GH followed by gonadotropins, TSH, and ACTH; this order is mainly seen in patients with tumors and after radiation therapy, while hypopituitarism due to other etiologies may present with a different sequence of deficiency, for example, ACTH deficiency may be the first manifestation in hypophysitis (1,22,30). Importantly, some patients may present with acute onset of pituitary hormone deficiency, while a dangerous presentation is the sudden emergence of ACTH deficiency (29).

Hypopituitarism may be associated with several metabolic and cardiovascular comorbidities, such as hypertension, unfavorable changes in body composition (with increased total and abdominal fat associated with decreased lean mass), and decreased exercise capacity, which may be accompanied by dyslipidemia, insulin resistance, premature atherosclerosis, and cardiac dysfunction (1,4-6). Insulin sensitivity varies greatly among patients with hypopituitarism, depending on numerous factors such as the etiology and treatment of the underlying cause, obesity, severity of pituitary deficiency, and inadequate hormone replacement (31). Metabolic syndrome, as defined by the National Cholesterol Education Program (NCEP) Adult Treatment Panel III criteria, has been observed in roughly 40% of the adult patients with hypopituitarism, and is associated with an increased risk of diabetes and vascular events (12,32-34).

Aside from the symptoms resulting from specific hormone deficiencies in hypopituitarism, the symptoms related to the underlying cause of hypopituitarism can dominate the clinical presentation. This is the case of pituitary tumors that induce visual changes, such as bitemporal hemianopsia due to compression of the optic chiasm, or diplopia due to invasion of the cavernous sinus and involvement of the cranial nerves crossing this sinus. Other symptoms associated with tumor growth and local invasion are headache and cerebrospinal fluid leakage. Also, in the case of functioning pituitary tumors, symptoms resulting from increased hormone secretion may coexist and predominate in the clinical presentation (1).

## CENTRAL HYPOTHYROIDISM (TSH DEFICIENCY)

Central hypothyroidism (CH) is defined by a decrease in thyroid hormone secretion secondary to insufficient TSH stimulation of a normal thyroid gland (35,36). Mechanisms responsible for CH include decreased number of functioning thyrotrophs, decreased synthesis and/or secretion of hypothalamic TRH, decreased TRH release to the pituitary, and decreased biologic activity of TSH. In most cases, CH is associated with other pituitary deficiencies. The signs and symptoms of CH are similar to those present in primary hypothyroidism, including somnolence, tiredness, mild weight gain, cold intolerance, constipation, dry skin, and bradycardia. The main differences of CH compared with primary hypothyroidism are usually the absence of goiter and reduced severity of the symptoms (35,36).

The laboratory diagnosis of CH is based on low serum free T4 (FT4) levels concomitant with a low or inappropriately normal TSH level (35,36). Less commonly, serum TSH may be mildly elevated, usually below 10 IU/L. The measurement of total T4 may replace the measurement of FT4 in the diagnosis of childhood-onset CH, but the accuracy of total T4 is poorer in adulthood-onset CH, as approximately 45% of these patients have total T4 within the normal reference range. Of note, FT4 levels remain within the low-normal range in approximately 18% of the patients with adulthood onset CH (37). The TRH test and serum T3 levels are not useful for diagnosing CH. In longitudinal follow-up, a decrease of more than 20% in FT4 levels should alert for an increased risk of CH, even when the values remain within the low-normal range (35-37).

The diagnosis of subclinical CH (normal FT4) is cumbersome and difficult to confirm by serum thyroid function markers. On Doppler echocardiography, abnormal presystolic time measurements have been demonstrated in patients with subclinical CH, but further studies are needed before recommendation of widespread use of this parameter (38). Other markers of thyroid hormone peripheral actions, such as serum cholesterol, sex–hormone-binding globulin (SHBG), carboxy-terminal telopeptide of type I collagen, osteocalcin (also known as bone gamma-carboxyglutamic acid [Gla]-containing protein), and IL-2 soluble receptor (sIL-2R) have also been used to better define subclinical CH, but thus far, they have not shown good accuracy (36).

The mainstay of hormone replacement in CH is levothyroxine (LT4). Replacement with T3 is currently not recommended due to lack of evidence showing clinical superiority to LT4 replacement and safety (39,40). In adult patients, the average dose of LT4 is 1.6 µg/kg/day but varies depending on the duration of the disease, number of additional pituitary deficiencies, and concomitant replacement with GH and/or estrogens (37,39-41). In most cases, LT4 treatment can be started at full dose, except in older individuals or in patients with cardiac or neurological diseases, in whom therapy should be initiated at lower doses and titrated up with caution. In these cases, symptoms and serum FT4 levels should be assessed at 6- to 8-week intervals during titration and every 6–12 months thereafter. Of note, evaluation of the adrenal axis is recommended, and if concomitant ACTH deficiency is present, LT4 replacement should only be initiated after glucocorticoid replacement due to the risk of adrenal crisis (30,35,36). Dose adjustments of LT4 may be necessary during concomitant treatment with GH in children, since GH replacement increases T4 to T3 conversion (41).

Levels of FT4 in the lowest tertile of the reference range have been associated with unfavorable metabolic profile in patients with CH. Thus, targeted serum FT4 levels during LT4 replacement should be into the upper half of the reference range, with blood samples collected before the daily LT4 intake (39,40,42). On the other hand, higher LT4 doses should also be avoided due to the increased risk of thyrotoxicosis symptoms, adrenal crisis, and osteoporosis (43). Measurement of TSH and T3 levels are usually unnecessary and not recommended during LT4 therapy in CH (30). However, low/undetectable TSH is expected in patients with CH on adequate thyroid hormone replacement, whereas a high T3 level indicates excessive thyroid hormone replacement requiring dose adjustments. LT4 requirements increase by 20% to 50% during pregnancy in patients with primary hypothyroidism, but patients with CH generally do not require a similar increase due to preservation of the thyroid response to the thyroid-stimulating effect of β-hCG (44).

## SECONDARY ADRENAL INSUFFICIENCY (ACTH DEFICIENCY)

Secondary adrenal insufficiency (SAI) may occur due to deficiency of ACTH or CRH in disorders of the pituitary or hypothalamus, resulting in decreased secretion of adrenal cortex steroids, mainly cortisol and dehydroepiandrosterone (DHEA) (45,46). SAI is one of the least frequent pituitary function alterations in most patients with hypopituitarism due to pituitary tumors, but it may be the initial presentation in other etiologies, such as in drug-induced hypophysitis (22,47). The prevalence of SAI after surgery for treatment of pituitary adenomas varies widely, while this condition may also develop years after radiotherapy (30). SAI increases the risk of morbidity and mortality in patients with hypopituitarism, since it predisposes patients to adrenal crisis in situations of acute stress and intercurrent illness (48).

**Figure 1:**
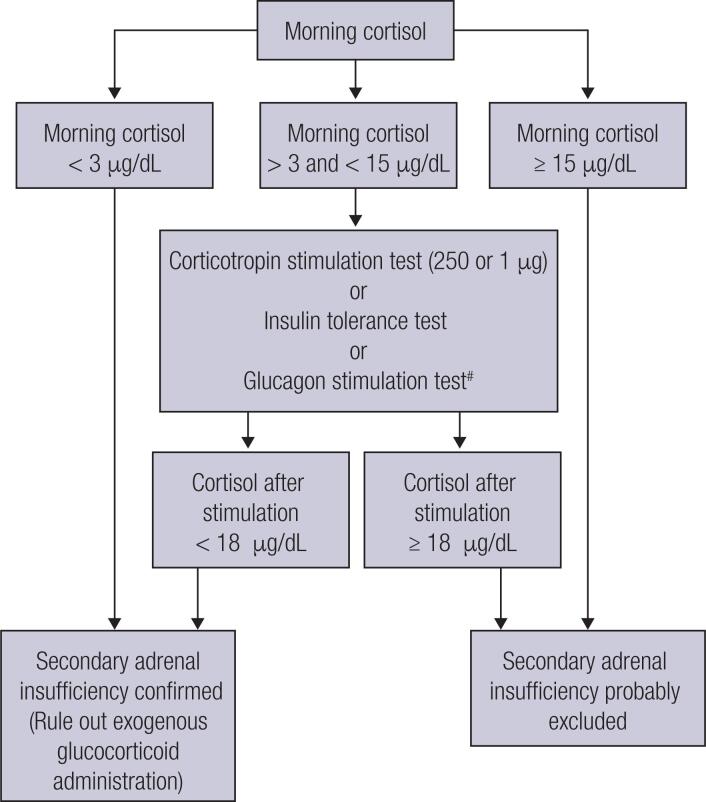
Hormonal investigation of secondary adrenal insufficiency (SAI)*

SAI manifests with nonspecific symptoms, such as nausea, dizziness, fatigue, anorexia, weight loss, and hypotension, thus requiring a high index of suspicion. Mild ACTH deficiency may only be clinically significant under concurrent stress or illness. The presence of normochromic normocytic anemia, eosinophilia, hyponatremia, hypoglycemia, and eventually hyperkalemia, should serve as a warning sign of increased risk of SAI in a patient with hypopituitarism (30,45,46). Nevertheless, long delays in diagnosing SAI are common, and in many patients, this condition is only established after hospitalization due to adrenal crisis, a potentially life-threatening medical condition requiring immediate emergency treatment. Adrenal crisis should be suspected in patients presenting with acute shock refractory to adequate fluid resuscitation and vasopressors (49). Estimates of yearly rates in patients with adrenal insufficiency in Europe project an incidence of adrenal crises of 5–10 per 100 patients and a mortality rate of 0.5 per 100 patient-years, corresponding to a number of deaths between 5,000–10,000 (49). Although a correct and prompt diagnosis of acute SAI is crucial for indicating appropriate therapy and preventing complications, the diagnostic investigation can be challenging in many cases, such as in critically ill patients (50). Importantly, diagnostic tests should never delay the prompt start of life-saving hydrocortisone treatment in suspected adrenal crisis (46). In the clinical context of hypopituitarism, the demonstration of morning cortisol levels collected at 8–9 AM lower than 100 nmol/L (3 μg/dL) are strongly predictive of SAI, whereas values greater than 15 μg/dL exclude this diagnosis. Of note, severely ill patients with SAI may present with morning cortisol levels greater than 500 nmol/L (18 μg/dL). Patients with morning cortisol values between 3–15 μg/dL require additional hormonal evaluation (30); in recommending such assessment, it is important to consider that normal individuals have morning cortisol values ranging from 6–15 μg/dL by most immunoassays (30,45,46). Despite considered the gold standard for diagnosis of SAI, the insulin tolerance test (ITT) requires adequate site and trained staff and is contraindicated in patients with epilepsy, cardiac arrhythmias, and cerebrovascular diseases. SAI is diagnosed if peak cortisol during ITT is lower than 500 nmol/L (18 μg/dL), although new immunoassay methods standardized against mass spectrometry can show lower concentrations, around 350 mmol/L (12 μg/dL). An alternative to ITT is the glucagon stimulation test (GST), which should be interpreted similarly to the ITT, but exhibits lower sensitivity and specificity (51). The simplest strategy to assess SAI is the short Synacthen test (or Cosyntropin test), in which serum cortisol levels are measured before and 30 and 60 minutes after stimulation with 250 μg of synthetic ACTH (52); a cortisol peak value below 500 nmol/L (< 18 µg/dL) confirms SAI. Unfortunately, Synacthen and Cosyntropin are not easily available in Brazil, hindering the use of these tests in clinical practice. The test may present false-negative results in SAI, especially within 4 weeks from a pituitary insult or surgery or in partial forms of the disease (46). A low-dose test (1 µg of synthetic ACTH) has been proposed, but has not shown any clear advantages compared with the traditional test (53) (Figure 1).

*The cut-off values of serum cortisol levels may change according to the method used for evaluation. ^#^ Cut-off value < 9,0 µg/dL.

In patients receiving glucocorticoid replacement, the exogenous glucocorticoid may suppress the hypothalamic-pituitary-adrenal axis and interfere with cortisol measurement. Patients using hydrocortisone can undergo serum cortisol assessment 24 hours after the last dose, but in those using prednisone or prednisolone, this period may be longer, requiring individual assessment based on the dose and the time of the last dose. These drugs may cross-react with cortisol measurement assays at different extents, and the duration of their suppression on the hypothalamic-pituitary-adrenal axis lacks consensus in the literature (54,55). Moreover, interference of oral estrogen should also be considered in the interpretation of the test results, since oral estrogen can increase serum cortisol levels by 20–50% due to increase in CBG concentrations, but does not affect salivary or urinary free cortisol measurements (56). There is no consensus in the literature about the diagnosis of complete or partial forms of SAI, which makes the diagnostic scenario even more complex. In this context, clinical judgment in each case is fundamental, and the general rule is to avoid excessive treatment. Plasma ACTH levels have no value in the diagnosis of SAI, and undetectable ACTH alone is unable to establish this diagnosis; this contrasts with a finding of elevated ACTH levels associated with low cortisol levels, which defines the diagnosis of primary adrenal insufficiency (Addison’s disease) (57). Other hormone alterations in patients with SAI include decreased or undetectable levels of DHEA and S-DHEA, particularly in younger patients. Women with panhypopituitarism and SAI have severe androgen deficiency, although the clinical importance of this finding is still unclear (46,57).

Once the diagnosis of SAI is confirmed, glucocorticoid replacement with one of the commercially available synthetic derivatives is recommended. Mineralocorticoid therapy is not necessary in SAI (30,46). Ideally, hydrocortisone should be the drug of choice for long-term SAI management, but the type of therapy and dosing regimen varies widely worldwide. In an international survey, 80% of the patients reported use of hydrocortisone, followed by prednisone/prednisolone (10%), cortisone acetate (3%), dexamethasone (3%), and other therapies (3%) (58). The daily dosage of hydrocortisone ranges between 15–20 mg divided into two to three doses; the largest dose should be taken upon awakening, followed by another dose close to lunchtime and, if needed, a third dose in the afternoon no later than 4–6 PM. Hydrocortisone and cortisone acetate are not widely available in Brazil, and the prescription of these salts by compounding pharmacies raises concerns about the origin, preservation, storage, packaging, and bioequivalence of these products and, therefore, are not recommended (30). Consequently, other formulations such as prednisone and prednisolone are more frequently prescribed. Prednisone is usually administered once daily in the morning, in doses ranging from 2.5–5.0 mg per day. Dose equivalence of hydrocortisone and prednisone can be calculated by the formula 1 mg prednisone = 4 mg hydrocortisone (59). Regardless of glucocorticoid type, replacement therapy in SAI should start with lower-range dosage regimens with up-titration according to the patient’s clinical status while avoiding overreplacement. During stress or intercurrent illness, patients should be instructed to double or triple the dose until resolution of the underlying condition. Patients, family members, and other health professionals should be educated about the correct adjustment of glucocorticoid dosage and informed on how to identify the precipitating factors and symptoms of adrenal crisis. In addition, patients should carry an identification and emergency card and be encouraged to use medical alert bracelets (45,46,49). In Brazil, educational material and emergency kits for patients with adrenal insufficiency are provided by the *Associação Brasileira Addisoniana* through their website at www.abaddison.org.br.

Modified-release hydrocortisone preparations have been developed to provide a more physiological replacement therapy, replicating the circadian rhythm of normal cortisol secretion (56). These compounds are formed by a dual-release formulation, consisting of one extended-release core surrounded by an immediate-release coating. One of these drugs, marketed as Plenadren, has been approved in Europe and seems to have some metabolic advantages over the conventional hydrocortisone (60). However, treatment cost is much higher with these drugs, and they have no perspective of receiving approval in Brazil.

There is no specific and accurate biomarker to guide glucocorticoid replacement, which leads clinicians to rely on subjective clinical assessments to adjust therapy. Not surprisingly, a real-world study of glucocorticoid replacement involving patients from the European Adrenal Insufficiency Registry (EU-AIR) identified 25 different regimens being used to deliver a daily hydrocortisone dose of 20 mg in clinical practice (61). Conflicting evidence has been shown regarding the effects of multiple treatment strategies on adverse health outcomes, such as quality of life (QoL), bone density, metabolic profile, and risk of adrenal crisis or death (59). However, prolonged use of doses higher than 20 mg of hydrocortisone or equivalent per day must be avoided as they have been associated with unfavorable metabolic profile, Cushing-like morbidities, and increased mortality in patients with hypopituitarism (62).

## HYPOGONADOTROPIC HYPOGONADISM (FSH/LH DEFICIENCY)

Hypogonadotropic hypogonadism (HH), also known as central or secondary hypogonadism, can be congenital or, more often, acquired. A detailed description of isolated congenital forms of HH is beyond the scope of this article (for a review on this topic, see Reference 63). Acquired HH is very common in patients with hypopituitarism due to pituitary adenomas and/or their treatment with surgery or radiotherapy, usually accompanied by other pituitary hormone deficiencies (12). Anorexia nervosa, excessive exercise, and psychological distress are common causes of acquired HH in women, and medications such as opiates, psychotropic agents, and glucocorticoids, can induce HH in both sexes. In adults, clinical features of HH in men include decreased libido, erectile dysfunction, and infertility, and in women include oligomenorrhea or amenorrhea, decreased libido, dyspareunia, and infertility that can be aggravated by concomitant hyperprolactinemia and adrenal/ovarian androgen deficiency (30). Prolonged estrogen deficiency may cause regression of secondary sexual characteristics, urinary symptoms, reduction of muscle mass and bone density, and dyslipidemia. Similarly, prolonged testosterone deficiency has been associated with an increased risk of metabolic disorders (64,65). Untreated HH is an independent factor related to high mortality due to vascular complications in patients with hypopituitarism, particularly women, and adequate replacement with sex steroids has been shown to normalize or reduce mortality rates in these patients (5,6,66).

In women, amenorrhea in the clinical context of a hypothalamic-pituitary disease indicates the presence of HH, which is confirmed by low estradiol levels associated with low or inappropriately normal FSH/LH levels (30). Similarly, low total testosterone levels associated with low or inappropriately normal FSH/LH levels establish the diagnosis of HH in men. Measurement of total testosterone should be performed before 10 AM after overnight fasting, in the absence of acute/subacute illness and medications (glucocorticoids, opiates, ketoconazole, barbiturates, cocaine, etc.) known to affect testosterone levels. Two measurements with the same type of assay are necessary to establish the diagnosis of low testosterone in cases of borderline values. There is no indication for stimulation tests (67).

### Sex steroid replacement therapy

Treatment of male HH involves primarily testosterone replacement, with the goal of improving sexual function, libido, energy levels, bone mineral density (BMD), sense of well-being, muscle mass, and strength. Current guidelines recommend the use of minimal doses necessary to maintain testosterone levels in the range of 450–600 ng/dL (30,67). Different testosterone formulations are available, and an individualized therapeutic approach is needed considering effectiveness, patient compliance and preference, drug cost and availability, and potential side effects (68-70).

Long-acting testosterone undecanoate is often considered the best option among injectable preparations due to its more convenient administration (intramuscular, every 10 to 14 weeks). This regimen usually maintains plasma testosterone levels in the expected range with minor fluctuations (68-70). Intramuscular formulations containing testosterone enanthate, cypionate, or the combination of four esters (propionate, phenylpropionate, isocaproate, and decanoate) are usually administered every 2 to 3 weeks, and are efficacious and generally well tolerated (71). Although these formulations are much less expensive than testosterone undecanoate, they are associated with supraphysiologic peak values shortly after the injection and to subphysiologic levels on the days before the new injection. This often leads to fluctuations in symptoms, mood swings, and emotional instability (71). Transdermal gel formulations are commonly prescribed, as they provide flexibility of dosing, ease of application, good skin tolerability, and possibly less erythrocytosis than injectable testosterone (68-70). The disadvantages of transdermal gel formulations include the potential of transferring of testosterone to women or children by direct skin-to-skin contact, and skin irritation in a small proportion of treated men (68-70). Testosterone undecanoate is the only safe testosterone ester for oral therapy, since it is well tolerated and not hepatotoxic, but it must be taken twice daily with fatty meals (68,72). It has been approved in the US market, but it is still not available in Brazil. Additionally, a nasal gel formulation and a subcutaneous testosterone enanthate formulation for weekly administration have been recently approved by the US Food and Drug Administration (FDA) (73,74) (Table 3). Testosterone therapy should not be started in men with breast or prostate cancer, palpable prostate nodule or induration, and prostate-specific antigen (PSA) > 4 ng/mL or > 3 ng/mL in those at increased risk of prostate cancer (e.g., men with a first-degree relative with diagnosed prostate cancer) without further urological evaluation, elevated hematocrit, untreated severe obstructive sleep apnea, severe lower urinary tract symptoms, uncontrolled heart failure, myocardial infarction or stroke within the last 6 months, or thrombophilia. After starting testosterone therapy, patients should be monitored with measurement of hematocrit and PSA levels in the first year of treatment to investigate possible side effects (67).

**Table 3:** Testosterone (T) formulations and commercial names (in parentheses)

Formulation	Dosage	Advantages	Disadvantages
Injectable long-acting T undecanoate in oil (Nebido, Hormus)	1000 mg IM, followed by 1000 mg at 6 weeks; then, 1000 mg every 10–14 weeks	Convenient drug regimen (once every 10-14 weeks); stable T levels	Requires IM injection of a large volume (3 or 4 mL); coughing (rarely); coughing episode immediately after injection (rarely); high cost
T enanthate (Delatestryl)*	100–200 mg IM every 2–4 weeks or 100 mg/week	Flexibility of dosing; low cost	Requires IM injection; peaks and valleys in serum T concentrations that may be associated with fluctuations in symptoms; coughing immediately after injection (very rarely)
T cypionate (Deposteron)	100–200 mg IM every 2–4 weeks or 100 mg/week		
T proprionate + isocaproate + decanoate + phenylpropionate (Durateston)	125–250 mg IM every 2–4 weeks or 125 mg/week		
Subcutaneous T enanthate (Xyosted)*	Starting dose: 75 mg subcutaneously once a week. The dose can be titrated to 50 mg or 100 mg weekly	Convenient drug regimen (once weekly); stable T levels	Increases in hematocrit, PSA, and blood pressure are the most frequent side effects
Transdermal T gel(Androgel)	50–100 mg of 1% transdermal gel once daily	Provides flexibility of dosing, ease of application, good skin tolerability; less erythrocytosis than injectable T	Potential of transfer to a female partner or child by direct skin-to-skin contact; T concentrations may be variable from application to application; skin irritation in a small proportion of patients
Axillary T solution (Axeron)	60 mg of T solution applied in the axillae	Provides good skin tolerability	Similar to 1% testosterone gel
Transdermal T patch (Androderm)*	One or two patches, designed to deliver 2–4 mg of T during 24 hours applied on a clean, dry area of skin on the arm, back, abdomen, or upper buttocks (once daily for most patients)	Ease of application; stable T levels	Serum T concentrations in some T-deficient men may be in the low-normal range; these men may need applications of two patches daily; skin irritation at the application site occurs frequently
Buccal, bioadhesive, T tablets (Striant)*	30 mg controlled release, bioadhesive tablets BID	Convenience and discreet	Twice daily applications are required. Gum-related adverse eventsin 16% of treated men; alterations in taste
T pellets (Testopel)*	T Pellets containing 600–1200 mg T implanted SC; the number of pellets and the regimen may vary with formulation	Requires infrequent administration	Surgical incision for insertions is required; occasional spontaneous pellets extrusion; local hematoma and infection rarely seen
Nasal T gel (Natesto)	11 mg two or three times daily	Rapid absorption and avoidance of first pass metabolism	Multiple daily intranasal dosing required; local nasal side-effects (rhinorrhea, epistaxis, nasal discomfort, nasal congestion, parosmia); not appropriate for men with nasal disorders
Oral T undecanoate (Jatenzo)*	Starting dose: 237 mg orally once in the morning and once in the evening (with meals). If needed, adjust the dose to a minimum of 158 mg BID and a maximum of 396 mg BID	Convenience of oral administration	Variable clinical responses; administration with fatty meal is required; fat content of meals affects bioavailability; variable serum T concentrations

* Not available in Brazil. IM: intramuscular; BID: twice daily; PSA: prostate-specific antigen. Adapted from Bhasin et al. Testosterone therapy in men with hypogonadism: an Endocrine Society Clinical practice guideline. J Clin Endocrinol Metab. 2018,103:1715-44.

Sex steroid replacement should be started in all women with HH who are at premenopausal age and be individualized for older women (30,68). Noteworthy, hypopituitarism removes the natural survival advantage that women have over men in terms of vascular complications; therefore, outcomes observed in natural menopause should not be extrapolated to women with hypopituitarism presenting with frank hypogonadism (75). In this regard, data of 203,767 postmenopausal women from 10 observational studies included in the International Collaboration for a Life Course Approach to Reproductive Health and Chronic Disease Events (InterLACE) indicated that primary hypogonadism due to surgical menopause was associated with over 20% higher risk of cardiovascular disease compared with natural menopause, with an increased risk in women younger than 40 years. Women who experienced surgical menopause at an earlier age (<50 years) and received hormone replacement therapy had a lower risk of incident heart disease than those who were not treated (76). Assuming that untreated FSH/LH insufficiency may have a negative rather than a beneficial effect on survival, it is unfortunate that HH is significantly more frequently underdiagnosed and undertreated in women compared with men (77,78).

While the goal of treatment of HH in older women is to control hot flushes, in young women, the goal is to prevent damage induced by estrogen deficiency. Adult women with HH should receive combined estrogen-progestogen regimen or unopposed estrogens (if prior hysterectomy) for improving symptoms of hypoestrogenism such as vaginal atrophy, dysuria, dry skin/hair, hot flushes, and night sweats, and to reduce the risk of cardiovascular disease, osteopenia, and mortality (30,68). The choice of estrogen and progestin formulations, route (oral or transdermal), and regimen depends on the risk of adverse effects, patient’s convenience/preference, and cost. Transdermal administration of estrogens either by patch or gel provides more stable plasma concentrations of estradiol and reduces its conversion to estrone compared with oral formulations. By eliminating the first pass through the liver, transdermal estrogen prevents undesirable increases in coagulation factors, blood pressure, and serum triglycerides. In addition, by interfering less with SHBG levels, transdermal estrogen allows a greater fraction of hormone to circulate in free and biologically active forms (68,79). The usual doses of transdermal estrogen are 50–100 µg of estradiol daily, applied as a patch twice weekly, or 1.5 mg daily of estradiol hemihydrate gel. The addition of progestogens to the patch does not affect the absorption of estrogen. Some application systems use patches containing varying amounts of estradiol that mimic the physiological variations of estradiol levels throughout the menstrual cycle. Some patients may develop allergy at the patch application site or exhibit a drop in estrogen levels earlier than expected (68). Furthermore, the transdermal route of estrogen administration does not block the hepatic generation of IGF-1 induced by GH (79).

For use in oral preparations, natural estradiol such as 17-beta-estradiol should be micronized or administered in derivative compounds such as ethinyl estradiol, or estradiol valerate, enanthate, or cypionate, to improve absorption. Benefits of oral estradiol therapy include relatively low cost, long accumulated experience, convenience, increased HDL-cholesterol, and reduced LDL-cholesterol (68,79). Another alternative to oral estradiol replacement are conjugated estrogens, a combination of estrogens obtained from urine of pregnant mares. The induction of protein synthesis in the liver by conjugated estrogens is even higher than that observed with the use of estradiol formulations (80). Daily doses of estrogen in adult women with HH range from 1.0 to 2.0 mg of micronized estradiol or an equivalent dose of other estrogens. In general, these doses lead to clinical improvement and serum estradiol levels between 30–50 pg/mL, which correspond to the levels observed in the early follicular phase (68,80). A Brazilian study evaluating bone mass in patients with premature ovarian failure has shown that the doses of estrogen normally used in postmenopause were not adequate to reduce impaired spinal and femoral bone mass in younger women (81), although no consensus in the literature has established the required estrogen dose in this age group.

Cyclic progesterone replacement combined with continuous estrogen prevents endometrial hyperplasia and induces menstruation. In this regimen, 2.5 to 10 mg of medroxyprogesterone acetate (MPA) or synthetic progestogenic derivative is added during 7 to 10 days of the month. Alternatively, estrogens and progestogens can be used simultaneously and continuously, which induces a higher frequency of amenorrhea (80). Alternatives to MPA are micronized progesterone (100–200 mg daily) and progestogens such as norethindrone (0.35 mg), gestodene (0.75 mg), or levonorgestrel (0.075 mg), which exhibit different affinities for the progesterone, testosterone, estradiol, and aldosterone receptors, and consequently have variable side effects (80). Endometrial proliferation should be controlled periodically by transvaginal ultrasonography. If endometrial thickening greater than 5 mm is observed, an endometrial biopsy should be performed (82).

Combined estrogen-progestin contraceptive pill may be more acceptable for younger women, but studies comparing the effects of different dose regimens in HH are lacking (30). After the age of physiological menopause, the prescription of estrogens should be based on current postmenopausal guidelines. Nevertheless, a recent systematic review comprising 2,588,327 postmenopausal women from six clinical trials and 27 prospective observational studies has observed that doses of 0.3–0.625 mg/day of oral conjugated equine estrogen or equivalent have been associated with cardioprotective effects, while higher doses increase the risk of venous thromboembolism and stroke in a dose-dependent manner. The authors concluded that hormone therapy should be used in the lowest effective dose to prevent adverse cardiovascular effects and that the dose should be reduced with advancing age (83). They also observed that transdermal estrogen may be safer in relation to cardiovascular and thrombotic risk than oral estrogen and that preparations with MPA are associated with increased thrombotic risk (83).

Women with HH have androgen deficiency. Although few studies have shown advantages with treatment (84), recent guidelines recommended against the routine use of DHEA and testosterone in women due to limited data concerning efficacy and safety (30,85,86). An intravaginal form of DHEA has been approved by the FDA to treat genitourinary syndrome of menopause, but this formulation is not available in Brazil (87). Moreover, testosterone has been carefully suggested in the guidelines to treat selected women with hypoactive sexual desire disorder (85-87). In this clinical context, which might be present in women with hypopituitarism, a trial of low-dose testosterone therapy may be considered, which seems to be safe in the short term. However, efficacy is variable and long-term effects on cardiovascular risk and breast cancer incidence are not known. Undesirable side effects, such as alopecia, changes in vocal timbre, hirsutism, clitoromegaly, acne, elevated hematocrit, and liver function and lipid profile abnormalities, must be balanced against the potential benefits (85-87).

### Fertility issues

Fertility issues in patients harboring aggressive pituitary tumors have been recently revisited (88). Importantly, fertility preservation strategies need to be discussed with the patient desiring conception before or during treatment. Additional pituitary deficiencies should be appropriately treated, particularly GH deficiency, which has been associated in some studies with poor pregnancy rates in patients with hypopituitarism (89). In men and women with HH, the approach to restoring fertility may include clomiphene citrate, human chorionic gonadotropin (hCG), human menopausal gonadotropin, or purified or recombinant FSH (88). Pulsatile GnRH administration via mini-infusion pump is only delivered in some research settings worldwide (63).

In men with fertility issues, testosterone replacement treatment should be discontinued. In men with prolactinomas and persistent hypogonadism under dopamine agonist therapy, clomiphene restores normal testosterone levels and improves sperm motility, independent of prolactin levels (90). In contrast, clomiphene treatment fails to restore normal testosterone levels in men with hypogonadism harboring nonfunctioning pituitary adenomas (91). A study reported that 67% of men with HH and acromegaly treated with clomiphene achieved normal testosterone levels, but fertility restoration was not evaluated (92). Administration of hCG is done intramuscularly or preferably subcutaneously in the thigh at an initial dose of 3,000–5,000 IU per week, divided into at least two injections to ensure relatively stable serum levels (63). Serum testosterone concentration is measured every 2 or 3 months, and the dose should be increased if the values are not between 400 and 800 ng/dL. Very rarely, serum testosterone concentration fails to respond to hCG, even at a very high dose, due to antibodies to hCG (93). If the sperm count does not reach 5–10 million/mL and/or pregnancy has not occurred within 6 months after serum testosterone is between 400–800 ng/dL, purified or recombinant FSH should be added to the regimen with a starting dose of 75–150 IU every other day (or three times per week). This dose is adjusted upwards if necessary to achieve serum FSH concentration in the physiologic range of 4–8 IU/L (63). These regimens induce testicular growth in almost all patients, spermatogenesis in approximately 80%, and pregnancy in 50% of the cases after 12–24 months of treatment (88).

In women, clomiphene citrate may induce ovulation in some infertile patients. Therapy with gonadotropin initiates in the follicular phase of the menstrual cycle with FSH, followed by administration of hCG. Ovulation is expected between 36 and 48 hours after hCG injection. If fertilization occurs, progesterone is administered to ensure implantation and is maintained until approximately 10 weeks of gestation, when placental hormone production becomes sufficient. Assisted reproduction technologies are alternative options when therapy with gonadotropins fails (88). In a recent systematic literature review, the authors suggested that assisted fertility in women hypopituitarism has a good outcome both in terms of achieving pregnancy and infant outcome (94).

## GH DEFICIENCY (GHD) IN ADULTS

A nationwide study carried out in Denmark has reported an annual incidence of adult GH deficiency (AGHD) of 17.6 per 1,000,000 people, with higher rates in men (95). Applying these numbers to the Brazilian population, we would expect approximately 3,700 new cases of AGHD yearly in our country. According to the age at onset, patients may present with childhood-onset (CO) AGHD or adulthood-onset AGHD (96-98). Isolated idiopathic GHD diagnosed in children based on biochemical GH tests must be reevaluated during transition into adult life. A Brazilian study reported that only 31% of the children with GHD persisted with GHD when retested, with an IGF-I cutoff value of 110 ng/mL presenting 94.5% sensitivity and 100% specificity for diagnosis of AGHD in the transition period (99). Noteworthy, short children treated with GH for non-GHD pediatric indications (e.g., Turner syndrome, idiopathic short stature, small for gestational age, etc.) and adults with functional GHD (obesity, metabolic syndrome, aging) without evidence of hypothalamic-pituitary disease must not be investigated for AGHD (100).

The clinical presentation of AGHD is characterized by multiple, nonspecific clinical findings involving changes in body composition, increased fracture risk, altered physical capacity, presence of cardiovascular risk factors, and poor QoL (101) (Table 4). These manifestations vary according to age, time of onset, and presence of other hormone deficiencies. For instance, low bone mass is more frequent in young adults with GHD, while cardiovascular risk factors are more common among older patients with GHD (102). In the United Kingdom, perceived impairment of QoL is required to initiate GH replacement in adults, while lack of improvement in QoL is used for discontinuation of GH therapy (103). QoL can be evaluated by general questionnaires or by the specific questionnaire Quality of Life Assessment of Growth Hormone Deficiency in Adults, which has been translated into Brazilian Portuguese and validated for the Brazilian population (104).

**Table 4:** Clinical abnormalities in adult patients with hypopituitarism and growth hormone (GH) deficiency (AGHD) and effect of GH replacement therapy

Clinical findings	Effects of AGHD	Effects of GH therapy
Body composition	Increased total/visceral fat	Reduced total/visceral fat
	Reduced lean body mass	Increased lean body mass
	Reduced bone mass (especially during transition and young adulthood)	Increased bone mass
	Increased fracture risk	Reduced bone fractures
Quality of life (QoL)	Fatigue (low energy, reduced vitality)	Improvement in women and patients with low QoL at baseline
	Low mood	
	Low self-esteem	
	Reduced concentration	
	Reduced memory	
	Increase in sick days	
	Greater social isolation	
Physical capacity	Reduced muscle strength	Improvement of muscle strength (long term), maximum VO_2_, cardiac function, and anaerobic capacity
	Reduced maximum oxygen uptake (VO_2_)	
	Impaired cardiac function	
	Reduced anaerobic capacity	
Cardiovascular risk factors	Dyslipidemia	Improvement in diastolic blood pressure, total cholesterol, low-density lipoprotein cholesterol, and endothelial functions Decreased inflammatory markers and intimal media thickening
	Hypertension	
	Abnormal fibrinolytic activity	
	Increased inflammatory markers	
	Increased intimal media thickening	
	Endothelial dysfunction	

**Figure 2:**
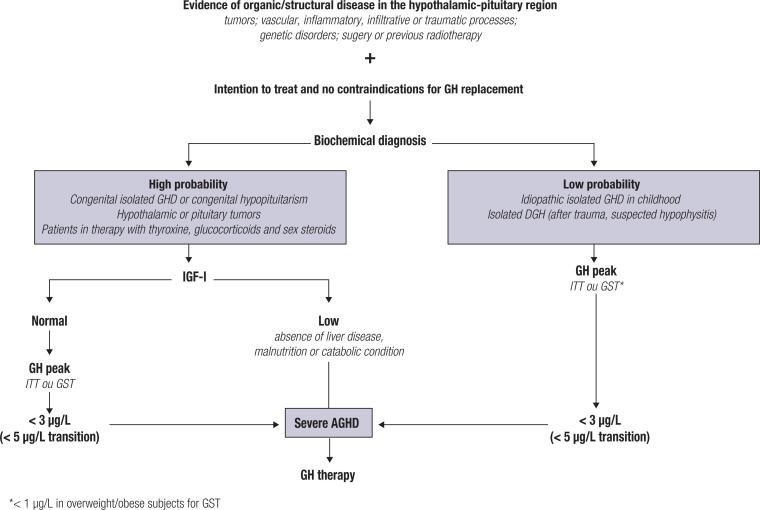
Algorithm for diagnosis of growth hormone (GH) deficiency in adult patients (AGHD) with hypopituitarism, considering only GH tests available in Brazil

ITT: insulin tolerance test; GST: glucagon stimulation test.

In an appropriate clinical context, the diagnosis of AGHD must be biochemically confirmed by serum IGF-I measurements alone or in association with GH stimulating tests. Basal serum GH levels have no diagnostic value due to the pulsatile nature of pituitary GH secretion. ITT is considered the gold-standard test, and severe GHD is diagnosed when the peak GH value is below 3 µg/L or 5 µg/L in the transition period (96-98,101,102). As mentioned before, ITT requires medical supervision and is contraindicated in elderly individuals and in patients with cardiac or neurologic diseases. An alternative to ITT is the GST, with a cutoff value of 3 µg/L recommended to discriminate between normal response and severe AGHD, or 1 µg/L in overweight/obese subjects (51). Macimorelin acetate is an orally active ghrelin mimetic that binds to the GH secretagogues receptors and stimulates GH secretion. The macimorelin test for AGHD has a sensitivity and specificity of 92% and 96%, respectively, with a diagnostic accuracy similar to that of ITT, without risk of hypoglycemia and less false-positive results. However, macimorelin acetate is an expensive medication and is not available in Brazil (101). Other GH tests commonly used in children to diagnose GHD are not recommended in adults due to very low accuracy (96-98). In patients with a high probability of AGHD, such as those with multiple pituitary hormone deficiencies associated with well-documented structural lesions in the hypothalamic-pituitary region, a low serum IGF-I level (below the lower limit of reference for age and sex) is enough to diagnose AGHD and precludes the need for a provocative GH test (96-98). However, factors interfering with IGF-I measurement should be considered, such as the use of oral estrogens, uncontrolled hypothyroidism, malnutrition, hepatic insufficiency, and uncontrolled diabetes, since these conditions may lead to false results. On the other hand, it is important to emphasize that normal IGF-I levels do not rule out the diagnosis of AGHD (96-98) (Table 5 and Figure 2).

**Table 5:** Biochemical tests available in Brazil for diagnosis of adult growth hormone (GH) deficiency (AGHD)

Test	Protocol	Diagnosis criteria	Advantages	Considerations
Insulin tolerance test (ITT)	0.1 U/kg IV of regular insulin; measure glucose and GH at baseline and 30, 60, 90, and 120 minutes after insulin	GH peak < 3 μg/L (< 5 μg/L in the transition period)	Gold standard May allow simultaneous assessment of hypothalamic-pituitary-adrenal axis	Risk of serious adverse events Requires supervision Contraindicated in elderly individuals and patients with neurologic or cardiac disorders
Glucagon stimulation test (GST)	Glucagon 1 mg IM (1.5 mg for patients > 90 kg); measure glucose and GH at baseline and every 30 minutes after glucagon for 3–4 hours	GH peak < 3 μg/L (< 1.0 μg/L in overweight/obese individuals)	Glucagon is readily available May allow simultaneous assessment of hypothalamic-pituitary-adrenal axis	Adverse effects (nausea, vomiting, headache) Requires better standardization of time points and cut-off values
IGF-I	None	Below the lower limit of normal (adjusted for age)	Simple	A normal value does not exclude AGHD

IV: intravenous; IM: intramuscular.

GH replacement therapy is indicated to all patients with a diagnosis of AGHD for whom treatment is intended (90). The main contraindications to GH therapy include active malignancy, uncontrolled diabetes, diabetic retinopathy, and intracranial hypertension (96-98). In Brazil, the Ministry of Health Ordinance number 28 of November 30, 2018, contemplates the “Clinical Protocol and Therapeutic Guidelines for Hypopituitarism”, a document that regulates in the public health care system (SUS) the therapeutic use of GH in children and adults (105). In contrast with GH treatment in children, GH replacement doses in adults are not calculated based on body weight, because this approach results in a high frequency of adverse events, mainly related to fluid retention, including paresthesias, joint stiffness, peripheral edema, arthralgia, myalgia, and carpal tunnel syndrome. Thus, the starting dose of GH is 0.1 mg for individuals 60 years or older, 0.2 mg for young men, 0.3 mg for young women, and 0.4–0.7 mg in the transition period, administered in daily subcutaneous injections at bedtime. The dose should be titrated every month until attaining IGF-I levels between the median and upper limit of normal for age (96-98). The maintenance dose generally varies between 0.4–0.6 mg/day, with a large individual variability depending on sex, age, body mass index (BMI), and route of estrogen replacement in women, among other factors (98). SAI and CH can develop or worsen during GH therapy; therefore, cortisol and FT4 should be closely monitored with proper adjustments in glucocorticoid and LT4 replacement doses (96-98,101,102). Anthropometry (BMI, waist circumference), blood pressure, and QoL should be assessed at each visit during follow-up for dose titration and every 6 months thereafter. Lipid profile, glycemia, and hemoglobin A1C can be evaluated every 6–12 months, while evaluation of body composition and BMD by dual-energy x-ray absorptiometry (DXA) and cardiovascular parameters is recommended yearly or every 2 years (96-98,101,102).

GH replacement therapy improves or normalizes several abnormalities related to AGHD and may promote health benefits in patients with hypopituitarism (Table 4). In the transition period, continuation or reintroduction of GH therapy after attaining final height promotes full somatic development of bone and muscles and prevents the development of abnormal metabolic features observed in older patients with AGHD (96,102). Meta-analyses of placebo-controlled trials have shown that GH treatment in adults reduces total and visceral fat mass, increases lean body mass and bone mass, improves cardiovascular risk markers (diastolic blood pressure, total cholesterol, and LDL-cholesterol), and increases anaerobic and exercise capacity (106,107). However, individual responses to GH therapy concerning different therapeutic endpoints vary greatly, and not all patients show the same pattern of improvement (108). It is still unclear whether GH replacement improves QoL, but in cohorts of AGHD followed up for up to 10 years, sustained improvement in QoL scores toward normal values has been demonstrated, especially in women and patients with low QoL at baseline (107,109).

GH replacement is generally safe and well tolerated in adult patients with hypopituitarism. However, special attention should be given to older, heavier, and female AGHD patients, as well as to those with a family history of type 2 diabetes, because they are more susceptible to adverse events, especially related to glucose homeostasis (110). In these individuals, close monitoring with fasting blood glucose and hemoglobin A1C is recommended during GH therapy. GH therapy should be initiated when pituitary tumor growth is under control. In this context, there is no evidence that long-term GH replacement in adults affects the progression of pituitary tumors. Additionally, there is no evidence of tumor recurrence or increased risk of neoplasia in AGHD; therefore, cancer surveillance in adult patients on GH therapy should follow the same standard practice as those in the general population (111).

The impact of untreated GHD on increasing mortality in patients with hypopituitarism is unclear. GH therapy does not change the prevalence of metabolic syndrome over time but, intriguingly, has been associated with reduction or even normalization of mortality in AGHD (6,32,33,112,113). A possible reason for this apparent paradox is the positive GH effect on endothelial and cardiovascular parameters (75). Analysis of a Brazilian kindred with congenital isolated GHD due to a homozygous mutation in the GH-releasing hormone (GHRH) receptor gene showed no serious deleterious effect of congenital isolated GHD on risks of vascular events and survival (114). However, this finding should not be extended to patients with hypopituitarism and AGHD, who present a distinct phenotype (115). Unfortunately, randomized clinical trials evaluating the effect of GH therapy on mortality in AGHD are unavailable. Thus, the primary goal of GH replacement therapy should be oriented to improve the associated morbidities in the short and long term.

## HORMONAL INTERACTIONS DURING PITUITARY HORMONE REPLACEMENT

Pituitary hypofunction usually requires replacement of several hormones, and it is essential to understand the main interactions between the different pituitary axes during hormone replacement to optimize the therapeutic outcomes. Prescription of thyroid hormones without previous replacement of glucocorticoids may precipitate an adrenal crisis in patients with ACTH deficiency. The mechanisms involved in cortisol reduction after LT4 treatment are not completely understood but may involve accelerated cortisol catabolism induced by an increase in renal 11β-hydroxysteroid dehydrogenase type 2 or increased urinary cortisol clearance due to augmented urinary flow caused by hypothyroidism treatment (68). Therefore, a careful investigation of SAI should be performed before initiating thyroid hormone therapy in patients with hypopituitarism, while the daily glucocorticoid dose may require adjustment in patients on glucocorticoid replacement who are started on LT4 treatment. Similarly, cortisol status must be monitored before and during GH replacement therapy, since GH reduces the activity of the 11β-hydroxysteroid dehydrogenase type 1 in the liver and adipose tissue, an enzyme that converts inactive cortisone to active cortisol (96-98). Consequently, GH therapy can unmask subclinical SAI, requiring the initiation or adequation of glucocorticoid replacement (96-98). In addition, patients replaced with GH can develop a variable reduction in FT4 levels and a slight increase in free T3 levels (41). The underlying mechanisms likely involve enhancement of peripheral deiodination of T4 to T3 due to activation of deiodinase type 1 by GH, reduction of residual TSH secretion due to an increase in somatostatinergic tonus determined by IGF-I action on hypothalamus, and/or reduction of residual TSH secretion due T3 feedback on the pituitary (116). Accordingly, FT4 levels should be evaluated 6 weeks after starting GH therapy, and LT4 initiation or dose adjustment may be necessary in patients with established CH to maintain FT4 levels in the adequate range (30).

**Figure 3:**
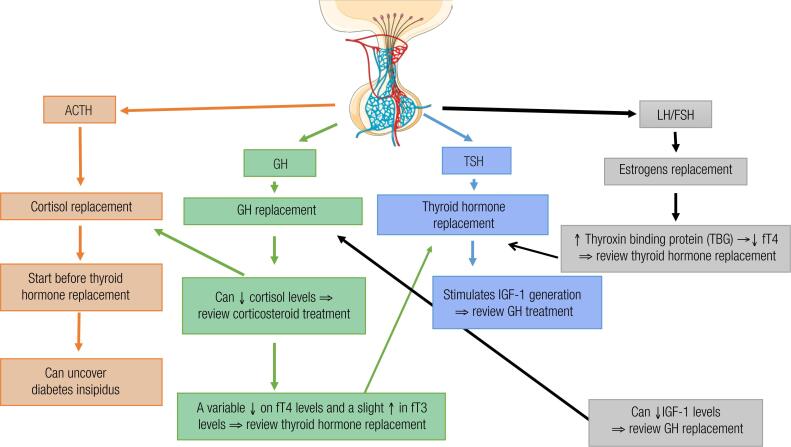
Pituitary hormone replacement and interactions.

Estrogen therapy may increase thyroxin binding protein (TBG) levels, reducing FT4 levels (117); consequently, the mean LT4 dose is usually higher in women on estrogen therapy than in those not taking estrogen (37). As previously mentioned, oral estrogen may antagonize GH action in the liver. This effect is induced by the expression of an intracellular protein named SOCS-2, which blocks GH signaling and reduces IGF-I generation (118). In addition, oral estrogens can interfere with IGFBPs levels during GH treatment. (119). Like oral estrogens, selective estrogen receptor modulators (SERMs) can also interfere with the hepatic IGF-I production (120). Thus, as previously mentioned, women with hypopituitarism and on GH replacement should receive estrogen via transdermal route. Taken together, all these interactions highlight the importance of constant clinical and laboratory monitoring of patients with hypopituitarism on replacement therapies to improve compliance, make appropriate adjustments, and optimize therapeutic outcomes (Figure 3).

fT4: free thyroxine; fT3: free triiodothyronine.

## FINAL COMMENTS

Management of hypopituitarism is one of the greatest challenges in clinical endocrinology, and several fundamental aspects for adequate control of this condition are presented in this document. Patients and family members must be educated to understand the particularities of hypopituitarism treatment and hormonal interactions. This is particularly important during clinical complications, surgeries, and any procedure that the patient may require. Physicians working in other specialties must also become knowledgeable in hormone replacement to be able to manage patients with hypopituitarism as needed.
